# Clinical and Molecular Characteristics of Human Rotavirus G8P[8] Outbreak Strain, Japan, 2014

**DOI:** 10.3201/eid2306.160038

**Published:** 2017-06

**Authors:** Kenji Kondo, Takeshi Tsugawa, Mayumi Ono, Toshio Ohara, Shinsuke Fujibayashi, Yasuo Tahara, Noriaki Kubo, Shuji Nakata, Yoshihito Higashidate, Yoshiki Fujii, Kazuhiko Katayama, Yuko Yoto, Hiroyuki Tsutsumi

**Affiliations:** Sapporo Medical University School of Medicine, Sapporo, Japan (K. Kondo, T. Tsugawa, M. Ono, Y. Yoto, H. Tsutsumi);; Tomakomai City Hospital, Tomakomai, Japan (T. Ohara);; Tomakomai Children's Clinic, Tomakomai (S. Fujibayashi);; Steel Memorial Muroran Hospital, Muroran, Japan (Y. Tahara);; Japanese Red Cross Urakawa Hospital, Urakawa, Japan (N. Kubo); Nakata Pediatric Clinic, Sapporo (S. Nakata);; Sapporo Hokushin Hospital, Sapporo (Y. Higashidate);; National Institute of Infectious Diseases, Tokyo, Japan (Y. Fujii, K. Katayama)

**Keywords:** rotavirus, G8P[8] rotavirus strains, genotype, outbreak, epidemic, vaccines, viruses, Asia, Japan, clinical features, molecular characterization, enteric infections

## Abstract

During March–July 2014, rotavirus G8P[8] emerged as the predominant cause of rotavirus gastroenteritis among children in Hokkaido Prefecture, Japan. Clinical characteristics were similar for infections caused by G8 and non-G8 strains. Sequence and phylogenetic analyses suggest the strains were generated by multiple reassortment events between DS-1–like P[8] strains and bovine strains from Asia.

Rotaviruses, the leading cause of acute gastroenteritis in children worldwide, are classified into G and P genotypes on the basis of 2 outer capsid proteins, viral protein (VP) 7 and VP4. A recently established extended rotavirus genotyping system based on the sequence of all 11 genome segments (*1*) grouped most human rotaviruses into 2 genotype constellations: Wa-like (G1/3/4/9-P[8]-I1-R1-C1-M1-A1-N1-T1-E1-H1) and DS-1–like (G2-P[4]-I2-R2-C2-M2-A2-N2-T2-E2-H2) strains.

In industrialized countries, rotavirus genotype G8 infection is common in bovines but rarely occurs in humans; however, the G8 strains are highly prevalent among humans in some countries in Africa (*2*). We investigated the clinical and molecular features of G8P[8] rotavirus, which, we unexpectedly found to be the predominant genotype in southwestern Hokkaido Prefecture, Japan, in 2014.

## The Study

**Figure 1:**
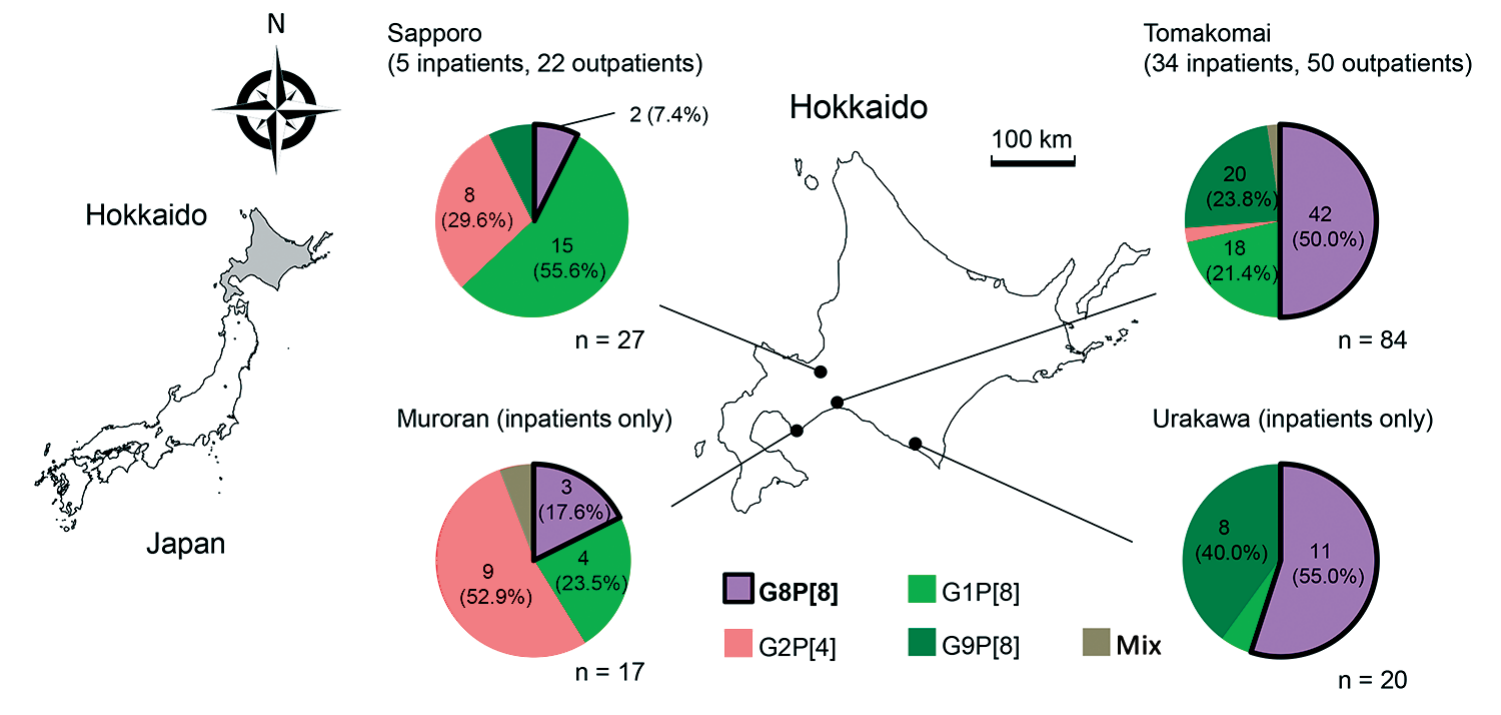
Distribution of rotavirus samples and their G/P genotypes in Hokkaido Prefecture (center map), Japan, 2014. The 4 locations from which the fecal samples were collected are shown on the map. Four hospitals (Tomakomai City Hospital, Japanese Red Cross Urakawa Hospital, Steel Memorial Muroran Hospital, and Sapporo Hokushin Hospital), and 2 clinics (Nakata Pediatric Clinic [Sapporo] and Tomakomai Children's Clinic) participated in the study. Map at left shows location of Hokkaido in Japan (gray shading).

During March–July 2014, we obtained rotavirus-positive fecal samples from 165 children in Hokkaido with acute gastroenteritis. The children were receiving care as inpatients or outpatients at 1 of 6 medical facilities (4 hospitals and 2 clinics) in the cities of Sapporo, Tomakomai, Muroran, and Urakawa (Figure 1).

For each fecal sample, we prepared a 10% fecal suspension, from which we extracted viral RNA. We performed reverse transcription PCR on the RNA by using the SuperScript II Reverse Transcriptase (Invitrogen, Carlsbad, CA, USA); PrimeSTAR GXL DNA polymerase (Takara, Shiga, Japan); and previously described primers (*3**,**4*). We used the BigDye Terminator v.3.1 Cycle Sequencing Reaction Kit (Applied Biosystems, Foster City, CA, USA) to sequence PCR amplicons. For some of the rotavirus samples, next-generation sequencing was performed at the National Institute of Infectious Diseases in Tokyo, Japan, as described previously (*5*). Sequences of the rotaviruses used in this study were submitted to the DDBJ under accession numbers LC102884–LC103134 and LC105000–LC105532. 

We successfully determined G and P genotypes for 148 of the 165 rotavirus samples by using the RotaC rotavirus genotyping tool (http://rotac.regatools.be/). The most common genotype was G8P[8], which was identified in 58 samples (39.2%), followed by G1P[8] (25.7%), G9P[8] (20.3%), and G2P[4] (12.8%) (Table 1; Technical Appendix). 

**Table 1:** Characteristics of patients infected with (G8P[8] and non-G8P[8] rotavirus, Tomakomai, Hokkaido Prefecture, Japan, 2014

Patient characteristic	Patients infected with rotavirus		p value*
	G8P[8], n = 42	Non-G8P[8], n = 42	
Sex, no.			1.0
M	24	24	
F	18	18	
Median age, y	1.8	2.0	0.50
Vaccinated, no (%)	2 (4.8)	1 (2.4)	0.56
Clinical symptoms, mean ± SD			
Duration of fever, d	1.3 ± 1.2	1.7 ± 1.4	0.13
Duration of diarrhea, d	2.4 ± 1.6	2.5 ± 3.2	0.51
Diarrhea episode in 24 h, maximum no.	3.6 ± 3.2	4.3 ± 4.2	0.69
Duration of vomiting, d	1.4 ± 1.2	1.8 ± 2.9	0.79
Vomiting episodes in 24 h, maximum no.	3.6 ± 3.8	3.7 ± 3.5	0.98
Admitted to hospital, no. (%)	18 (42.9%)	16 (38.1%)	0.66

*Categorical data were analyzed by using χ^2^ tests; continuous data were analyzed by using Student’s *t*-tests or Mann-Whitney U tests.

We obtained clinical data for all 84 patients who sought care at the hospital or clinic in Tomakomai. Demographic and clinical characteristics (e.g., age, sex, history of rotavirus vaccination, duration of fever, and duration and frequency of diarrhea and vomiting) were not substantially different between 42 patients with G8P[8] rotavirus infection and 42 patients with non-G8P[8] rotavirus infection. The proportion of patients admitted to hospitals was also similar in the 2 groups (Table 1).

We selected 15 G8P[8] strains for whole-genome analysis. All strains had the same genotype constellation, G8-P[8]-I2-R2-C2-M2-A2-N2-T2-E2-H2, indicating a genomic backbone of the DS-1 genotype constellation. The genomes of these G8P[8] strains shared >99.6% nt identity with each other (Table 2; Technical Appendix). All 11 genome segments of strain To14-0 (the representative G8P[8] strain in this study) exhibited the highest nucleotide identity to human G8P[8] strains isolated in Southeast Asia in 2014 (represented by strain RVN1149 from Vietnam [>99.4% nt identity] and NP-130 from Thailand [>99.5% nt identity]) (*6**,**7*) (Table 2). This finding suggests that the strains share a common G8P[8] origin.

**Table 2:** Genotype constellations and nucleotide identities of strains closely related to To14-0, the representative G8P[8] rotavirus strain used in a study of the clinical and molecular features of a G8P[8] rotavirus outbreak strain, Hokkaido Prefecture, Japan, 2014

Strain (genotype representative from study/country)	Genotype constellations and nucleotide identities (%), by gene*										
	VP7	VP4	VP6	VP1	VP2	VP3	NSP1	NSP2	NSP3	NSP4	NSP5
Human/To14-0 (G8P[8] in study)	G8 (**100**)	P[8] (**100**)	I2 (**100**)	R2 (**100**)	C2 (**100**)	M2 (**100**)	A2 (**100**)	N2 (**100**)	T2 (**100**)	E2 (**100**)	H2 (**100**)
Human/VNM/RVN1149/2014/G8P[8] (G8P[8] in Vietnam)	G8 (**99.4**)	P[8] (**99.8**)	I2 (**99.8**)	R2 (**99.6**)	C2 (**99.6**)	M2 (**99.8**)	A2 (**100**)	N2 (**99.9**)	T2 (**99.5**)	E2 (**99.9**)	H2 (**99.5**)
Human/THA/NP-130/2014/G8P[8] (G8P[8] in Thailand)	G8 (**99.7**)	P[8] (**99.9**)	I2 (**99.9**)	R2 (**99.9**)	C2 (**99.8**)	M2 (**99.9**)	A2 (**99.9**)	N2 (**99.7**)	T2 (**99.8**)	E2 (**99.7**)	H2 (**99.5**)
Human/THA/KKL-17/2013/G8P[8] (G8P[8] in Thailand)	G8 (**99.5**)	P[8] (**99.8**)	I2 (91.5)	R2 (**99.9**)	C2 (**99.9**)	M2 (**99.9**)	A2 (**99.9**)	N2 (**99.8**)	T2 (**99.9**)	E2 (**99.7**)	H2 (**99.2**)
Human/THA/LS-04/2013/G2P[8] (DS-1–like G2P[8] in Thailand)	G2 (–)	P[8] (**99.8**)	I2 (**99.5**)	R2 (86.0)	C2 (**99.8**)	M2 (97.4)	A2 (**99.9**)	N2 (**99.8**)	T2 (98.6)	E2 (95.5)	H2 (**99.7**)
Human/THA/SKT-109/2013/G1P[8] (DS-1–like G1P[8] in Thailand)	G1 (–)	P[8] (**99.7**)	I2 (**98.7**)	R2 (86.1)	C2 (**99.9**)	M2 (**99.8**)	A2 (**99.8**)	N2 (85.8)	T2 (**99.8**)	E2 (94.5)	H2 (**99.2**)
Human/To14-41 (DS-1–like G1P[8] in study)	G1 (–)	P[8] (98.8)	I2 (98.3)	R2 (86.3)	C2 (**99.4**)	M2 (**99.4**)	A2 (**99.5**)	N2 (85.5)	T2 (**99.6**)	E2 (93.5)	H2 (98.8)
Human/JPN/NT004/2012/G1P[8] (DS-1–like G1P[8] in Japan)	G1 (–)	P[8] (**99.0**)	I2 (98.4)	R2 (86.3)	C2 (**99.7**)	M2 (**99.5**)	A2 (**99.5**)	N2 (85.6)	T2 (**99.6**)	E2 (94.5)	H2 (**99.2**)
Human/THA/NP-M51/2013/G2P[4] (G2P[4] in Thailand)	G2 (–)	P[4] (–)	I2 (**99.5**)	R2 (86.0)	C2 (**99.1**)	M2 (97.5)	A2 (**99.0**)	N2 (85.4)	T2 (98.7)	E2 (95.5)	H2 (**99.7**)
Human/KOR/CAU15–11/2015/G2P[4] (G2P[4] in South Korea)	G2 (–)	P[4] (–)	I2 (**99.5**)	R2 (85.9)	C2 (**99.2**)	M2 (97.4)	A2 (94.0)	N2 (84.8)	T2 (98.1)	E2 (95.3)	H2 (98.6)
Human/MU14114 (G2P[4] in study)	G2 (–)	P[4] (–)	I2 (97.0)	R2 (85.3)	C2 (96.9)	M2 (86.4)	A2 (95.8)	N2 (85.5)	T2 (96.7)	E2 (95.6)	H2 (98.3)
Human/ITA/PR457/2009/G10P[14]	G10 (–)	P[14] (–)	I2 (93.0)	R2 (98.1)	C2 (82.3)	M2 (86.6)	A11 (–)	N2 (86.4)	T6 (–)	E2 (94.3)	H3 (–)
Caprine/BGL/GO34/1999/G6P[1]	G6 (–)	P[1] (–)	I2 (94.9)	R2 (86.3)	C2 (83.4)	M2 (86.8)	A11 (–)	N2 (86.6)	T6 (–)	E2 (96.0)	H3 (–)
Human/COD/DRC88/2003/G8P[8] (G8P[8] in Africa)	G8 (86.0)	P[8] (98.3)	I2 (96.3)	R2 (85.6)	C2 (97.4)	M2 (97.0)	A2 (96.4)	N2 (85.8)	T2 (97.1)	E2 (90.1)	H2 (97.4)
Human/MWI/QEC287/2006/G8P[8] (G8P[8] in Africa)	G8 (85.5)	P[8] (98.3)	I2 (96.3)	R2 (85.6)	C2 (97.8)	M2 (96.0)	A2 (96.5)	N2 (86.9)	T2 (96.6)	E2 (89.2)	H2 (97.6)
Human/MWI/QEC289/2006/G8P[8] (G8P[8] in Africa)	G8 (85.5)	P[8] (97.5)	I2 (96.3)	R2 (85.5)	C2 (97.8)	M2 (95.9)	A2 (96.5)	N2 (86.9)	T2 (96.6)	E2 (89.2)	H2 (97.6)

*Purple indicates G8 genotype; green indicates Wa-like genome segments; red indicates DS-1–like genome segments; bold indicates nucleotide identities >99.0%; – indicates percentages not calculated because the genotype was different that for To14-0.

**Figure 2:**
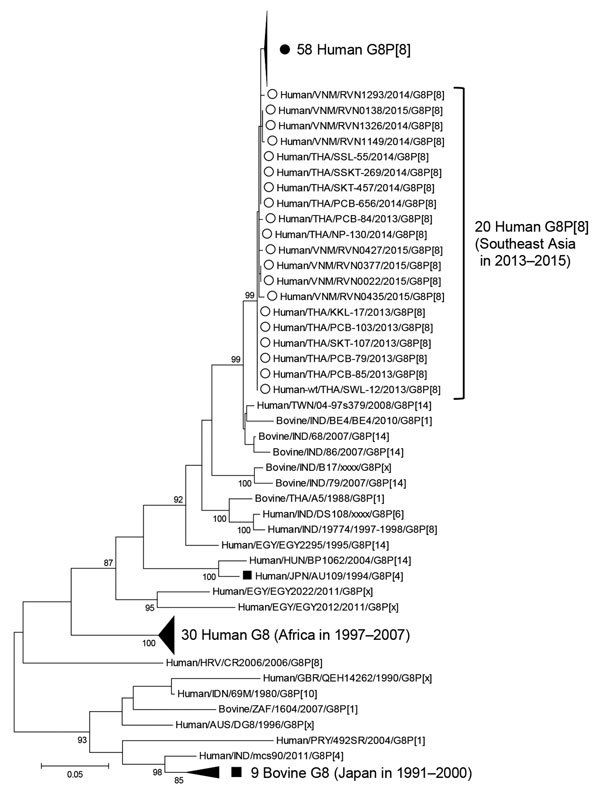
Phylogenetic analysis of the viral protein 7 gene of G8 rotavirus strains used in a study of the clinical and molecular features of a G8P[8] rotavirus outbreak strain, Hokkaido Prefecture, Japan, 2014. Closed circle indicates the G8P[8] rotavirus strain from Hokkaido; open circles indicate human G8P[8] strains from Southeast Asia; and closed boxes indicate other strains from Japan. A Tamura 3-parameter model was used for the maximum-likelihood method. Bootstrap values are shown at the branch nodes (values of <80% are not shown). Scale bar indicates nucleotide substitutions per site.

The VP7 gene of rotavirus strain To14-0 shared the highest nucleotide identity with the VP7 genes of human G8P[8] strains from Southeast Asia, including strains RVN1149 and NP-130 (99.4% and 99.7% identity, respectively), and it shared slightly lower identity to the VP7 gene of human strain 04-97s379 (97.8%) from Taiwan, which is speculated to be of bovine origin (*8*) (Figure 2). The VP7 genes of other G8 strains isolated in Japan were more distantly related to the To14-0 VP7 gene (e.g., human AU109 and bovine strains shared 89.5% and 81.9%–85.1%, respectively, with To14-0) (*9*). The VP7 genes of the human G8 strains prevailing in Africa were also distantly related (<90% nt identity) to the VP7 gene of To14-0.

Among the 11 To14-0 genome segments, 8 (VP2*–*VP4, VP6, nonstructural protein [NSP] 1*–*3, and NSP5) were highly similar to those of the human DS-1–like P[8] strains that have been isolated in Asia since 2012 (e.g., SKT-109, NT004, and LS-04) (*10*–*12*), including the strains isolated in this study (e.g., To14-41) (Table 2; Technical Appendix, panels B–H, J). In addition, the VP6 and NSP5 genes of the strains isolated in this study were also highly similar to those of human G2P[4] strains circulating in South Korea (strain CAU15-11) and Thailand (strain NP-M51) (*12*).

In contrast, the VP1 and NSP4 genes of To14-0 were only distantly related to those of the DS-1–like P[8] strains isolated in Asia (e.g., SKT-109, NT004, and LS-04), including the strains isolated in this study (e.g., To14-41) (Table 2; Technical Appendix Figure, panels A and I). The To14-0 VP1 gene shared high nucleotide identity with the VP1 genes of human G10P[14] strain PR457 from Italy (98.1%), which are probably the result of independent zoonotic transmissions (*13*). The To14-0 NSP4 gene shared high nucleotide identity with the NSP4 genes of human strains BSGH38 from India (96.7%) and the caprine G6P[1] strain GO34 from Bangladesh (96.0%) (*14*).

## Conclusions

The clinical characteristics recorded for patients infected with G8P[8] rotaviruses and those infected with non-G8P[8] rotaviruses did not differ (Table 1). Our findings suggest that the severity of gastroenteritis caused by newly emerging G8P[8] rotaviruses could possibly be attenuated by 1) the existence of VP7/VP4 genotype cross-reactive (heterotypic) protective responses; 2) protective immunity associated with other segments, such as VP6 and NSP4 (*3**,**15*); or 3) both of these factors combined.

The VP7 genes of the human G8P[8] strains isolated in this study and in Southeast Asia appear to have a close relationship with bovine strains from Asia but not from Japan, and the VP7 gene of human G8 or bovine G8 strains previously isolated in Japan are distantly related to them. Therefore, the VP7 genes in the G8P[8] strains from this study may have originated from a bovine strain from Asia. As with the VP7 genes, the VP1 and NSP4 genes are also assumed to have been derived from artiodactyl strains.

Eight genome segments (VP2*–*VP4, VP6, NSP1*–*NSP3, and NSP5) of the human G8P[8] strains isolated in this study and from Southeast Asia are closely related to those of the DS-1–like P[8] strains that have emerged and spread in Japan and other countries of Asia since 2012 (Table 2). Therefore, these 8 genome segments of the G8P[8] strains from this study may be derived from the DS-1–like P[8] strains in Asia.

For the reasons we have stated, the G8P[8] strains isolated in this study were speculated to be formed outside of Japan by multiple reassortment events between the DS-1–like P[8] strains and bovine strains in Asia. The resulting strain was probably recently introduced into Japan.

The predominance of novel DS-1–like G8P[8] strains noted in this study indicates that these strains are sufficiently adapted to humans to sustain human-to-human transmission in an industrialized country. This finding suggests that these G8P[8] rotavirus strains could spread to other regions in the near future. Continuing surveillance is required to monitor the circulating wild-type strains, and rotavirus genotype constellations and clinical information must be analyzed to understand rotavirus virulence in humans.

Technical Appendix Distribution of rotavirus G/P genotype combinations detected in Hokkaido Prefecture, Japan, in 2014; nucleotide identities at each genome segment of G8P[8] strains from this study compared with To14-0; and phylogenetic trees of genes from strains sequenced in this study and from reference strains.

## References

[1] Matthijnssens J, Ciarlet M, McDonald SM, Attoui H, Bányai K, Brister JR, et al. Uniformity of rotavirus strain nomenclature proposed by the Rotavirus Classification Working Group (RCWG). Arch Virol. 2011;156:1397–413. 10.1007/s00705-011-1006-z 21597953 PMC3398998

[2] Nakagomi T, Doan YH, Dove W, Ngwira B, Iturriza-Gómara M, Nakagomi O, et al. G8 rotaviruses with conserved genotype constellations detected in Malawi over 10 years (1997-2007) display frequent gene reassortment among strains co-circulating in humans. J Gen Virol. 2013;94:1273–95. 10.1099/vir.0.050625-0 23407423 PMC3945219

[3] Fujii Y, Shimoike T, Takagi H, Murakami K, Todaka-Takai R, Park Y, et al. Amplification of all 11 RNA segments of group A rotaviruses based on reverse transcription polymerase chain reaction. Microbiol Immunol. 2012;56:630–8. 10.1111/j.1348-0421.2012.00479.x 22708835

[4] Tsugawa T, Tatsumi M, Tsutsumi H. Virulence-associated genome mutations of murine rotavirus identified by alternating serial passages in mice and cell cultures. J Virol. 2014;88:5543–58. 10.1128/JVI.00041-14 24599996 PMC4019127

[5] Masuda T, Nagai M, Yamasato H, Tsuchiaka S, Okazaki S, Katayama Y, et al. Identification of novel bovine group A rotavirus G15P[14] strain from epizootic diarrhea of adult cows by de novo sequencing using a next-generation sequencer. Vet Microbiol. 2014;171:66–73. 10.1016/j.vetmic.2014.03.009 24725447 PMC7127257

[6] Hoa-Tran TN, Nakagomi T, Vu HM, Do LP, Gauchan P, Agbemabiese CA, et al. Abrupt emergence and predominance in Vietnam of rotavirus A strains possessing a bovine-like G8 on a DS-1-like background. Arch Virol. 2016;161:479–82. 10.1007/s00705-015-2682-x 26586330

[7] Tacharoenmuang R, Komoto S, Guntapong R, Ide T, Sinchai P, Upachai S, et al. Full genome characterization of novel DS-1–like G8P[8] rotavirus strains that have emerged in Thailand: reassortment of bovine and human rotavirus gene segments in emerging DS-1–like intergenogroup reassortant strains. PLoS One. 2016;11:e0165826. 10.1371/journal.pone.0165826 27802339 PMC5089778

[8] Wu FT, Bányai K, Wu HS, Yang DC, Lin JS, Hsiung CA, et al. Identification of a G8P[14] rotavirus isolate obtained from a Taiwanese child: evidence for a relationship with bovine rotaviruses. Jpn J Infect Dis. 2012;65:455–7. 10.7883/yoken.65.455 22996226 PMC8211372

[9] Agbemabiese CA, Nakagomi T, Doan YH, Nakagomi O. Whole genomic constellation of the first human G8 rotavirus strain detected in Japan. Infect Genet Evol. 2015;35:184–93. 10.1016/j.meegid.2015.07.033 26275468

[10] Komoto S, Tacharoenmuang R, Guntapong R, Ide T, Haga K, Katayama K, et al. Emergence and characterization of unusual DS-1–like G1P[8] rotavirus strains in children with diarrhea in Thailand. PLoS One. 2015;10:e0141739. 10.1371/journal.pone.0141739 26540260 PMC4634990

[11] Fujii Y, Nakagomi T, Nishimura N, Noguchi A, Miura S, Ito H, et al. Spread and predominance in Japan of novel G1P[8] double-reassortant rotavirus strains possessing a DS-1-like genotype constellation typical of G2P[4] strains. Infect Genet Evol. 2014;28:426–33. 10.1016/j.meegid.2014.08.001 25111613

[12] Komoto S, Tacharoenmuang R, Guntapong R, Ide T, Tsuji T, Yoshikawa T, et al. Reassortment of human and animal rotavirus gene segments in emerging DS-1–like G1P[8] rotavirus strains. PLoS One. 2016;11:e0148416. 10.1371/journal.pone.0148416 26845439 PMC4742054

[13] Medici MC, Tummolo F, Bonica MB, Heylen E, Zeller M, Calderaro A, et al. Genetic diversity in three bovine-like human G8P[14] and G10P[14] rotaviruses suggests independent interspecies transmission events. J Gen Virol. 2015;96:1161–8. 10.1099/vir.0.000055 25614586

[14] Ghosh S, Alam MM, Ahmed MU, Talukdar RI, Paul SK, Kobayashi N. Complete genome constellation of a caprine group A rotavirus strain reveals common evolution with ruminant and human rotavirus strains. J Gen Virol. 2010;91:2367–73. 10.1099/vir.0.022244-0 20505013

[15] Burns JW, Siadat-Pajouh M, Krishnaney AA, Greenberg HB. Protective effect of rotavirus VP6-specific IgA monoclonal antibodies that lack neutralizing activity. Science. 1996;272:104–7. 10.1126/science.272.5258.104 8600516

