# Supplementary material for: Clinical and Molecular Characteristics of Human Rotavirus G8P[8] Outbreak Strain, Japan, 2014
Source: Emerg Infect Dis. 2017 Jun;23(6):968–72. doi: 10.3201/eid2306.160038 (PMC5443423; doi:10.3201/eid2306.160038)
Supplement: Technical Appendix — Distribution of rotavirus G/P genotype combinations detected in Hokkaido Prefecture, Japan, in 2014; nucleotide identities at each genome segment of G8P[8] strains from this study compared with To14-0; and phylogenetic trees of genes from strains sequenced in this study and from reference strains. [file 16-0038-Techapp-s1.pdf]

Article DOI: https://dx.doi.org/10.3201/eid2306.160038

Clinical and Molecular Characteristics of Human Rotavirus G8P[8] Outbreak Strain, Japan, 2014

Technical Appendix

Technical Appendix Table 1. Distribution of G/P genotype combinations detected in Hokkaido, Japan in 2014

City	No. (%) typeable sample	G8P[8]	G1P[8]	G9P[8]	G2P[4]	Mixed*
Tomakomai	84	42 (50.0)	18 (21.4)	20 (23.8)	2 (2.4)	2 (2.4)
Urakawa	20	11 (55.0)	1 (5.0)	8 (40.0)	0	0
Muroran	17	3 (17.6)	4 (23.5)	0	9 (52.9)	1 (5.9)
Sapporo	27	2 (7.4)	15 (55.6)	2 (7.4)	8 (29.6)	0
Total	148	58 (39.2)	38 (25.7)	30 (20.3)	19 (12.8)	3 (2.0)

*Mixed infections: Tomakomai, G1P[8]+G9P[8]; Muroran, G2P[4]+G9P[8].

Technical Appendix Table 2. Nucleotide identities (%) at each genome segment of G8P[8] strains from this study compared with To14-0 (the representative G8P[8] strain in this study)

Location, strain name	VP7	VP4	VP6	VP1	VP2	VP3	NSP1	NSP2	NSP3	NSP4	NSP5
Tomakomai											
To14-0	100	100	100	100	100	100	100	100	100	100	100
To14-2	99.9	100	100	99.9	100	99.9	100	100	99.9	100	100
To14-14	99.9	99.9	99.9	100	100	100	100	100	99.9	100	100
To14-36	99.8	100	100	99.9	100	99.9	99.9	100	99.9	99.8	99.7
TKC14-6	99.9	99.6	100	100	99.9	100	100	99.8	99.9	100	100
TKC14-16	99.9	99.6	99.9	99.8	99.9	100	99.8	100	99.8	99.9	99.7
TKC14-37	99.9	99.6	100	99.9	100	99.9	100	100	99.8	99.9	100
Muroran											
MU14-0	99.9	99.6	99.9	100	100	99.9	100	99.9	99.9	99.9	100
MU14-2	99.9	99.9	100	99.9	100	100	99.9	99.9	99.8	99.7	100
MU14-3	99.9	99.6	99.9	99.7	100	99.9	100	100	99.9	100	99.6
Urakawa											
UR14-10	99.9	100	100	100	100	100	100	100	99.9	100	100
UR14-14	99.9	100	99.8	100	100	100	99.9	100	99.9	100	99.9
UR14-29	99.9	99.9	99.8	99.9	100	99.8	99.9	99.9	99.9	100	100
Sapporo											
N14-22	99.9	100	100	100	100	100	99.9	99.9	100	100	100
HK14-2	99.9	99.7	99.7	99.9	100	100	99.9	99.8	99.9	100	100

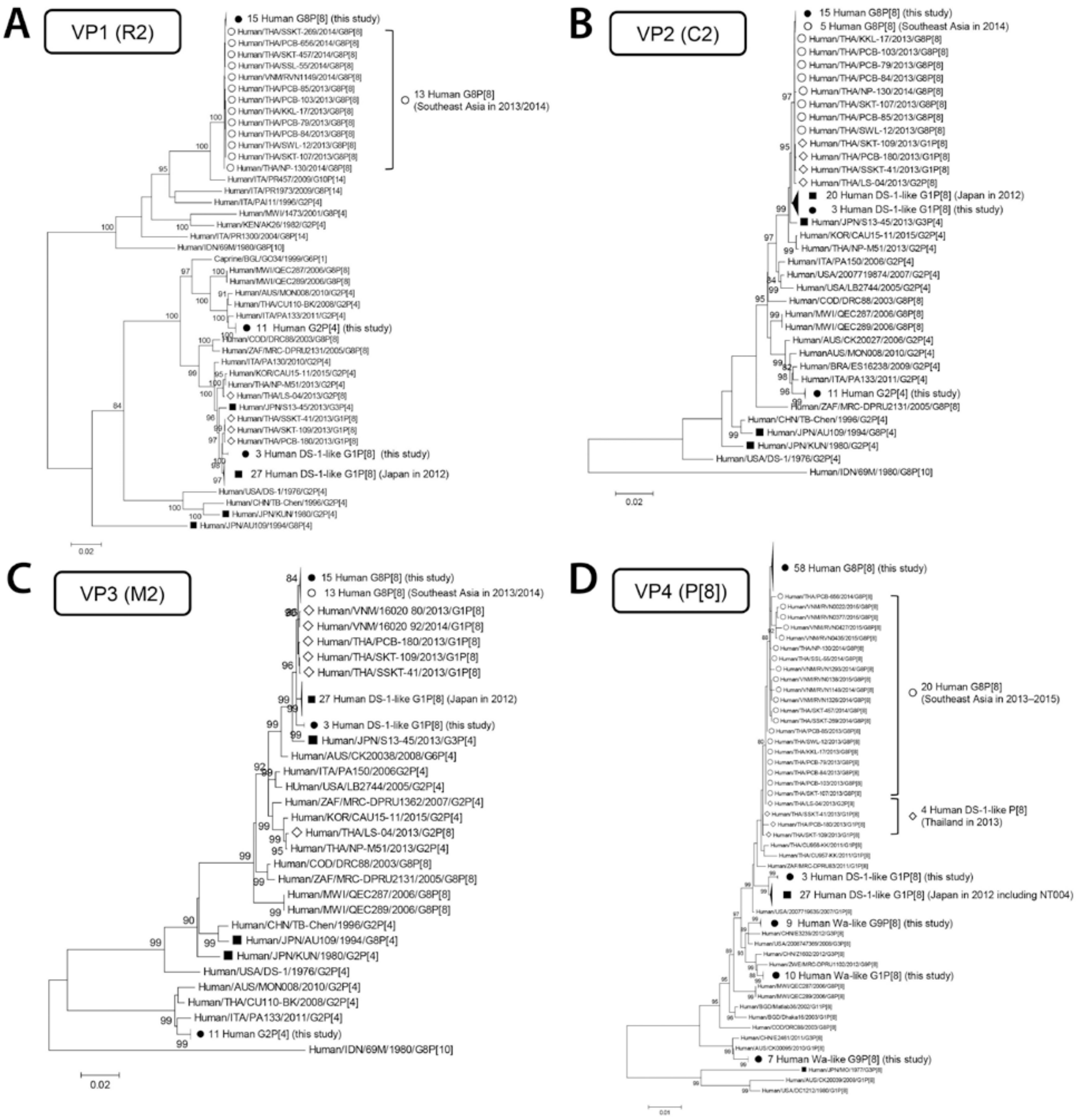
A
VP1 (R2)
15 Human G8P[8] (this study)
13 Human G8P[8] (Southeast Asia in 2013/2014)
11 Human G2P[4] (this study)
3 Human DS-1-like G1P[8] (this study)
27 Human DS-1-like G1P[8] (Japan in 2012)
B
VP2 (C2)
15 Human G8P[8] (this study)
5 Human G8P[8] (Southeast Asia in 2014)
20 Human DS-1-like G1P[8] (Japan in 2012)
3 Human DS-1-like G1P[8] (this study)
11 Human G2P[4] (this study)
Human/IDN/69M/1980/G8P[10]
0.02
C
VP3 (M2)
15 Human G8P[8] (this study)
13 Human G8P[8] (Southeast Asia in 2013/2014)
Human/VNM/16020 80/2013/G1P[8]
Human/VNM/16020 92/2014/G1P[8]
Human/THA/PCB-180/2013/G1P[8]
Human/THA/SKT-109/2013/G1P[8]
Human/THA/SSKT-41/2013/G1P[8]
27 Human DS-1-like G1P[8] (Japan in 2012)
3 Human DS-1-like G1P[8] (this study)
Human/JPN/S13-45/2013/G3P[4]
Human/AUS/CK20038/2008/G6P[4]
Human/ITA/PA150/2006G2P[4]
HUman/USA/LB2744/2005/G2P[4]
Human/ZAF/MRC-DPRU1362/2007/G2P[4]
Human/KOR/CAU15-11/2015/G2P[4]
Human/THA/LS-04/2013/G2P[8]
Human/THA/NP-M51/2013/G2P[4]
Human/COD/DRC88/2003/G8P[8]
Human/ZAF/MRC-DPRU2131/2005/G8P[8]
Human/MWI/QEC287/2006/G8P[8]
Human/MWI/QEC289/2006/G8P[8]
Human/CHN/TB-Chen/1996/G2P[4]
Human/JPN/AU109/1994/G8P[4]
Human/JPN/KUN/1980/G2P[4]
Human/USA/DS-1/1976/G2P[4]
Human/AUS/MON008/2010/G2P[4]
Human/THA/CU110-BK/2008/G2P[4]
Human/ITA/PA133/2011/G2P[4]
11 Human G2P[4] (this study)
Human/IDN/69M/1980/G8P[10]
0.02
D
VP4 (P[8])
58 Human G8P[8] (this study)
20 Human G8P[8] (Southeast Asia in 2013–2015)
4 Human DS-1-like P[8] (Thailand in 2013)
3 Human DS-1-like G1P[8] (this study)
27 Human DS-1-like G1P[8] (Japan in 2012 including NT004)
9 Human Wa-like G9P[8] (this study)
10 Human Wa-like G1P[8] (this study)
7 Human Wa-like G9P[8] (this study)

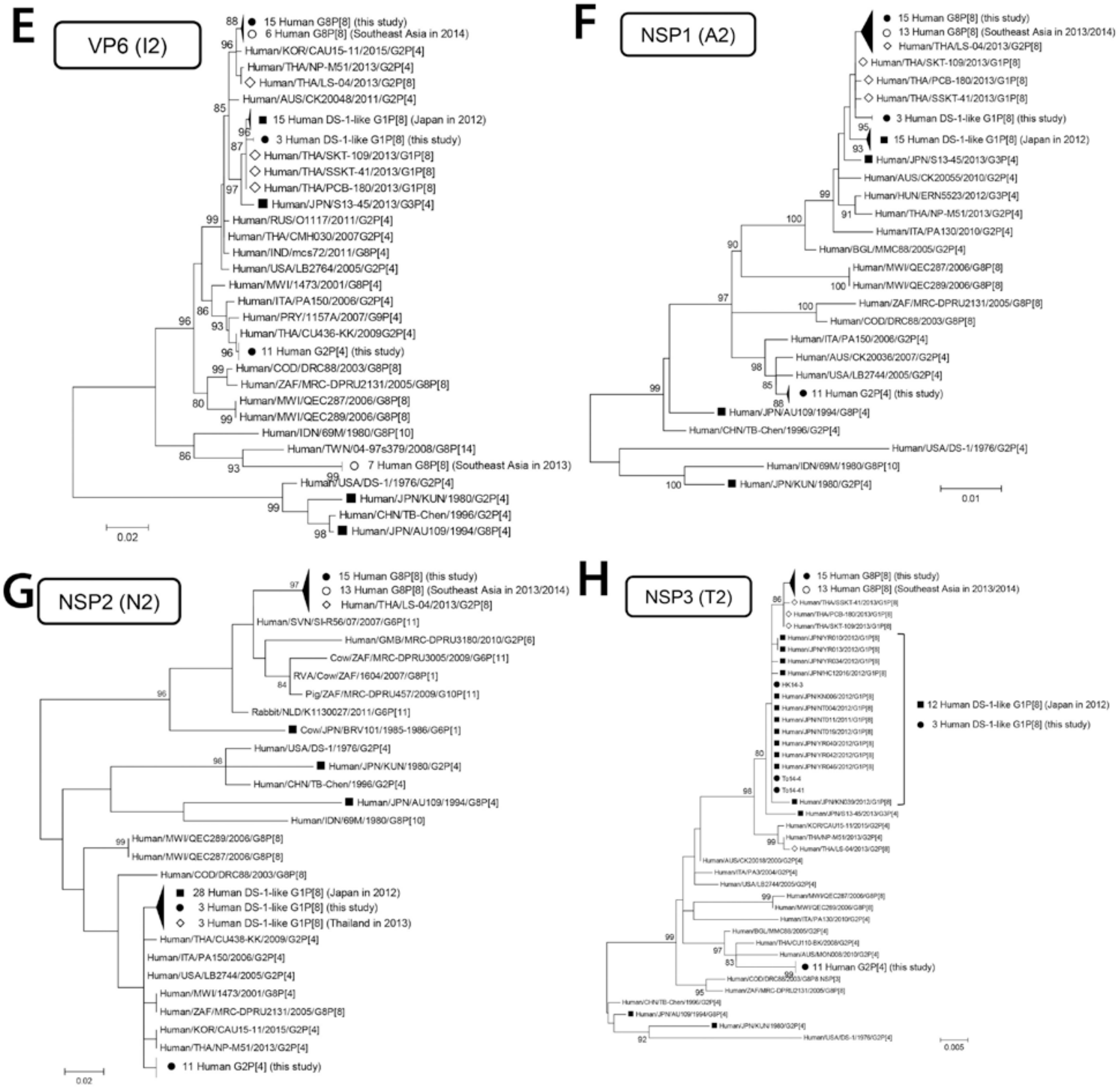
E
VP6 (I2)
15 Human G8P[8] (this study)
6 Human G8P[8] (Southeast Asia in 2014)
Human/KOR/CAU15-11/2015/G2P[4]
Human/THA/NP-M51/2013/G2P[4]
Human/THA/LS-04/2013/G2P[8]
Human/AUS/CK20048/2011/G2P[4]
15 Human DS-1-like G1P[8] (Japan in 2012)
3 Human DS-1-like G1P[8] (this study)
Human/THA/SKT-109/2013/G1P[8]
Human/THA/SSKT-41/2013/G1P[8]
Human/THA/PCB-180/2013/G1P[8]
Human/JPN/S13-45/2013/G3P[4]
Human/RUS/O1117/2011/G2P[4]
Human/THA/CMH030/2007G2P[4]
Human/IND/mcs72/2011/G8P[4]
Human/USA/LB2764/2005/G2P[4]
Human/MWI/1473/2001/G8P[4]
Human/ITA/PA150/2006/G2P[4]
Human/PRY/1157A/2007/G9P[4]
Human/THA/CU436-KK/2009G2P[4]
11 Human G2P[4] (this study)
Human/COD/DRC88/2003/G8P[8]
Human/ZAF/MRC-DPRU2131/2005/G8P[8]
Human/MWI/QEC287/2006/G8P[8]
Human/MWI/QEC289/2006/G8P[8]
Human/IDN/69M/1980/G8P[10]
Human/TWN/04-97s379/2008/G8P[14]
7 Human G8P[8] (Southeast Asia in 2013)
Human/USA/DS-1/1976/G2P[4]
Human/JPN/KUN/1980/G2P[4]
Human/CHN/TB-Chen/1996/G2P[4]
Human/JPN/AU109/1994/G8P[4]
0.02
F
NSP1 (A2)
15 Human G8P[8] (this study)
13 Human G8P[8] (Southeast Asia in 2013/2014)
Human/THA/LS-04/2013/G2P[8]
Human/THA/SKT-109/2013/G1P[8]
Human/THA/PCB-180/2013/G1P[8]
Human/THA/SSKT-41/2013/G1P[8]
3 Human DS-1-like G1P[8] (this study)
15 Human DS-1-like G1P[8] (Japan in 2012)
Human/JPN/S13-45/2013/G3P[4]
Human/AUS/CK20055/2010/G2P[4]
Human/HUN/ERN5523/2012/G3P[4]
Human/THA/NP-M51/2013/G2P[4]
Human/ITA/PA130/2010/G2P[4]
Human/BGL/MMC88/2005/G2P[4]
Human/MWI/QEC287/2006/G8P[8]
Human/MWI/QEC289/2006/G8P[8]
Human/ZAF/MRC-DPRU2131/2005/G8P[8]
Human/COD/DRC88/2003/G8P[8]
Human/ITA/PA150/2006/G2P[4]
Human/AUS/CK20036/2007/G2P[4]
Human/USA/LB2744/2005/G2P[4]
11 Human G2P[4] (this study)
Human/JPN/AU109/1994/G8P[4]
Human/CHN/TB-Chen/1996/G2P[4]
Human/USA/DS-1/1976/G2P[4]
Human/IDN/69M/1980/G8P[10]
Human/JPN/KUN/1980/G2P[4]
0.01
G
NSP2 (N2)
15 Human G8P[8] (this study)
13 Human G8P[8] (Southeast Asia in 2013/2014)
Human/THA/LS-04/2013/G2P[8]
Human/SVN/SI-R56/07/2007/G6P[11]
Human/GMB/MRC-DPRU3180/2010/G2P[6]
Cow/ZAF/MRC-DPRU3005/2009/G6P[11]
RVA/Cow/ZAF/1604/2007/G8P[1]
Pig/ZAF/MRC-DPRU457/2009/G10P[11]
Rabbit/NLD/K1130027/2011/G6P[11]
Cow/JPN/BRV101/1985-1986/G6P[1]
Human/USA/DS-1/1976/G2P[4]
Human/JPN/KUN/1980/G2P[4]
Human/CHN/TB-Chen/1996/G2P[4]
Human/JPN/AU109/1994/G8P[4]
Human/IDN/69M/1980/G8P[10]
Human/MWI/QEC289/2006/G8P[8]
Human/MWI/QEC287/2006/G8P[8]
Human/COD/DRC88/2003/G8P[8]
28 Human DS-1-like G1P[8] (Japan in 2012)
3 Human DS-1-like G1P[8] (this study)
3 Human DS-1-like G1P[8] (Thailand in 2013)
Human/THA/CU438-KK/2009/G2P[4]
Human/ITA/PA150/2006/G2P[4]
Human/USA/LB2744/2005/G2P[4]
Human/MWI/1473/2001/G8P[4]
Human/ZAF/MRC-DPRU2131/2005/G8P[8]
Human/KOR/CAU15-11/2015/G2P[4]
Human/THA/NP-M51/2013/G2P[4]
11 Human G2P[4] (this study)
0.02
H
NSP3 (T2)
15 Human G8P[8] (this study)
13 Human G8P[8] (Southeast Asia in 2013/2014)
12 Human DS-1-like G1P[8] (Japan in 2012)
3 Human DS-1-like G1P[8] (this study)
11 Human G2P[4] (this study)
0.005

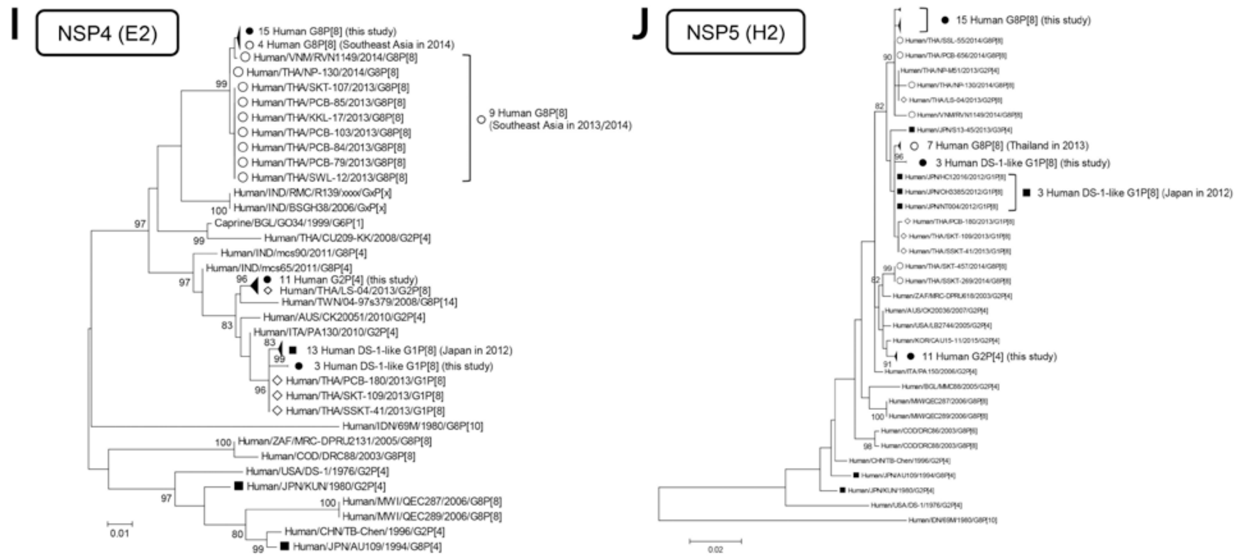

Technical Appendix Figure. Phylogenetic analyses of rotavirus genome segments. Phylogenetic trees of the VP1–3 (A–C), VP4 (D), VP6 (E), NSP1–5 (F–J) genes of the human strains sequenced in this study (closed circles) and other reference strains. The human Southeast Asian G8P[8] strain, the human Southeast Asian DS-1-like P[8] strains, and the Japanese strains are indicated by an open circle, open diamonds, and closed boxes, respectively. A Tamura 3-parameter model was used for the maximum-likelihood method. Bootstrap values are shown at the branch nodes (values of <80% are not shown). The scale bars indicate genetic distance.
